# Value of strain analysis with feature tracking in adenosine stress myocardial perfusion magnetic resonance imaging

**DOI:** 10.1186/1532-429X-16-S1-P193

**Published:** 2014-01-16

**Authors:** Christopher Schneeweis, Bernhard Schnackenburg, Alexander Berger, Sebastian Kelle, Eckart Fleck, Rolf Gebker

**Affiliations:** 1 Cardiology, German Heart Institute Berlin, Berlin, Germany; 2 Philips Research Hamburg, Hamburg, Germany

## Background

Adenosine stress magnetic resonance is highly accurate for the detection of myocardial perfusion abnormalities. Visual assessment of inducible wall motion abnormalities (IWMA) during adenosine has low sensitivity but high specificity since only high-grade perfusion defects are associated with detectable IWMAs. The novel technique Feature Tracking (FT) measures myocardial strain and gives detailed information about regional myocardial deformation. The aim of this study was to investigate FT for the detection of myocardial ischemia during adenosine stress.

## Methods

A total of 62 patients with suspected or known coronary artery disease (CAD) underwent adenosine stress CMR at 1.5 or 3T. Patients with evidence of myocardial scar iby late gadolinium enhancement (LGE), WMA at rest and impaired left ventricular function (LVEF) were excluded (n = 34). Short axis (SAX) cine views were acquired before and after 3 minutes of continuous adenosine infusion (0.14 mg/min/kg). Patients with abnormal stress perfusion were considered positive for myocardial ischemia (n = 15) and segments with perfusion defects were defined as ischemic (n = 61). Patients without perfusion defects and without known CAD were defined as normal and served as control (13 patients, 208 segments). TomTec 2D Cardiac Performance Analysis software was used to derive quantitative assessment of circumferential strain (CS) and strain rate (SR) from the three SAX cine views at rest and under stress.

## Results

**Figure 1:**
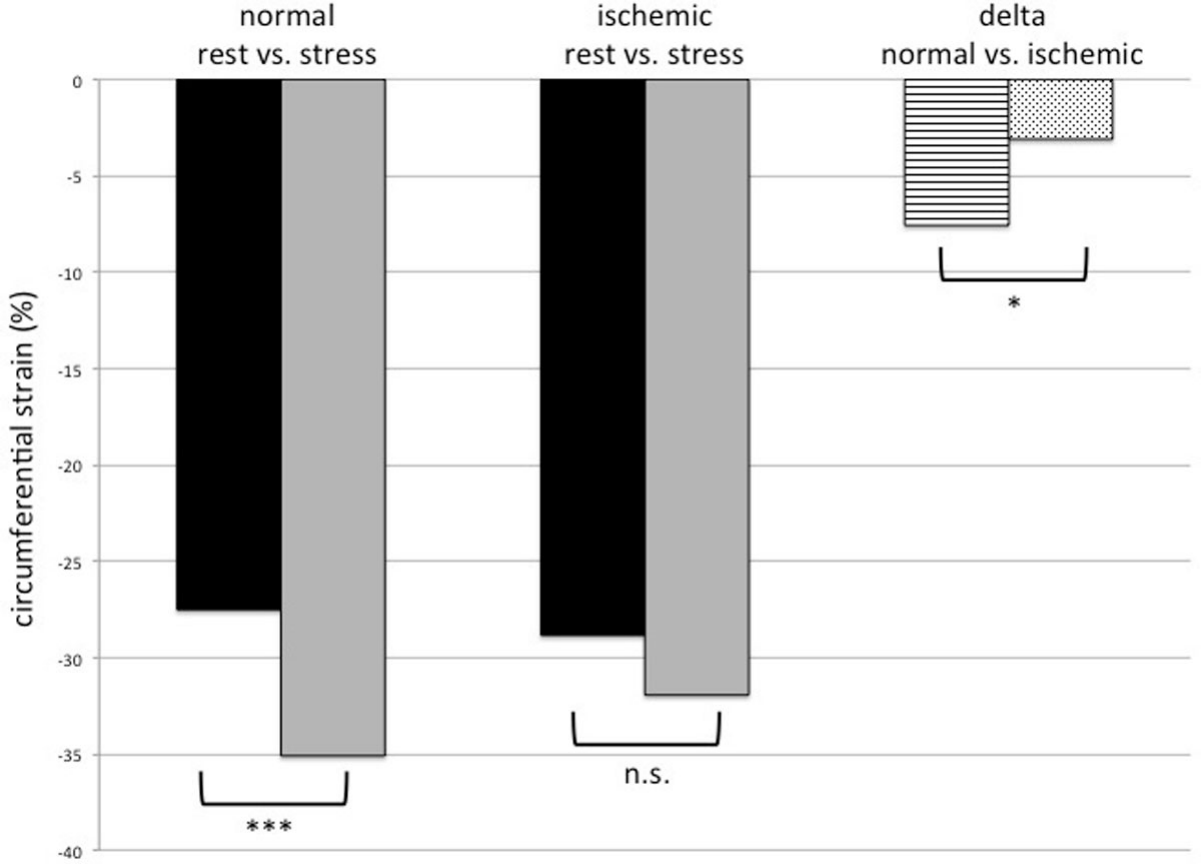


**Figure 2:**
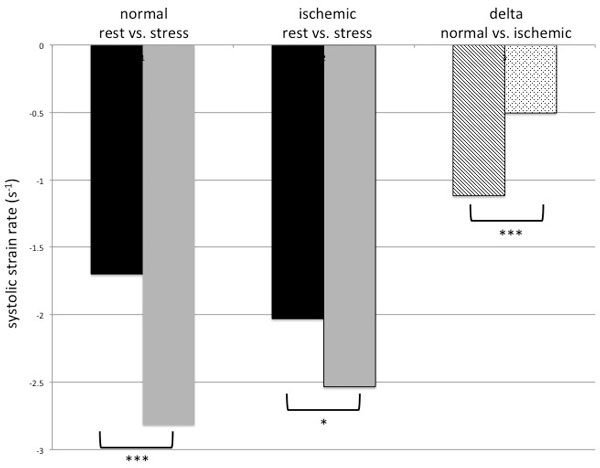


There was no significant difference of CS at rest between normal and ischemic segments (normal: -27.5 ± 9.1%; ischemic: -28.8 ± 9.6%, p = 0.32). Normal segments demonstrated a significant increase during stress for CS (-27.5 ± 9.1% vs. -35.1 ± 9.1%, p < 0.001, respectively) as well as for SR (1.7 ± 0.7 vs. 2.8 ± 1.4 s-1, p < 0.001, respectively). In ischemic segments no significant difference was observed for CS between rest and stress (-28.8 ± 9.6% vs. 31.9 ± 12.8%, p = 0.064; Figure 1), but for SR (rest 2.0 ± 0.8 vs. stress 2.5 ± 1.6 s-1, p = 0.013; Figure 2). The absolute differences in CS between rest and stress (Δ-CS) in ischemic segments were significantly smaller than Δ-CS in normal segments (-3.1 ± 12.8 vs. 7.6 ± 8.6, respectively, p = 0.017). The same observation occurred for Δ-SR (normal: 1.1 ± 1.2; ischemic: 0.5 ± 1.5, p < 0.001). The intraclass correlation coeffecient showed good agreement between two observers (CS rest: 0.82; CS stress: 0.85; SR rest: 0.76; SR stress: 0.79).

## Conclusions

Strain analysis with FT during adenosine stress demonstrated significant differences between normal and ischemic segments. Therefore it may be a valuable additional tool for the assessment of myocardial ischemia.

## Funding

No funding.

